# Wearable Multi-Functional Sensing Technology for Healthcare Smart Detection

**DOI:** 10.3390/mi13020254

**Published:** 2022-02-02

**Authors:** Xu Zeng, Hai-Tao Deng, Dan-Liang Wen, Yao-Yao Li, Li Xu, Xiao-Sheng Zhang

**Affiliations:** 1 School of Electronic Science and Engineering, University of Electronic Science and Technology of China, Chengdu 611731, China; zengxu_uestc@163.com (X.Z.); 2019110222337@std.uestc.edu.cn (H.-T.D.); dlwen@std.uestc.edu.cn (D.-L.W.); Liyaoyao_work@163.com (Y.-Y.L.); 2 Rehabilitation Department, Sichuan Provincial People’s Hospital, Chengdu 610072, China

**Keywords:** self-powered microsystems, multi-functional sensors, sensing mechanisms, health monitoring, wearable electronics

## Abstract

In recent years, considerable research efforts have been devoted to the development of wearable multi-functional sensing technology to fulfill the requirements of healthcare smart detection, and much progress has been achieved. Due to the appealing characteristics of flexibility, stretchability and long-term stability, the sensors have been used in a wide range of applications, such as respiration monitoring, pulse wave detection, gait pattern analysis, etc. Wearable sensors based on single mechanisms are usually capable of sensing only one physiological or motion signal. In order to measure, record and analyze comprehensive physical conditions, it is indispensable to explore the wearable sensors based on hybrid mechanisms and realize the integration of multiple smart functions. Herein, we have summarized various working mechanisms (resistive, capacitive, triboelectric, piezoelectric, thermo-electric, pyroelectric) and hybrid mechanisms that are incorporated into wearable sensors. More importantly, to make wearable sensors work persistently, it is meaningful to combine flexible power units and wearable sensors and form a self-powered system. This article also emphasizes the utility of self-powered wearable sensors from the perspective of mechanisms, and gives applications. Furthermore, we discuss the emerging materials and structures that are applied to achieve high sensitivity. In the end, we present perspectives on the outlooks of wearable multi-functional sensing technology.

## 1. Introduction

**Figure 1:**
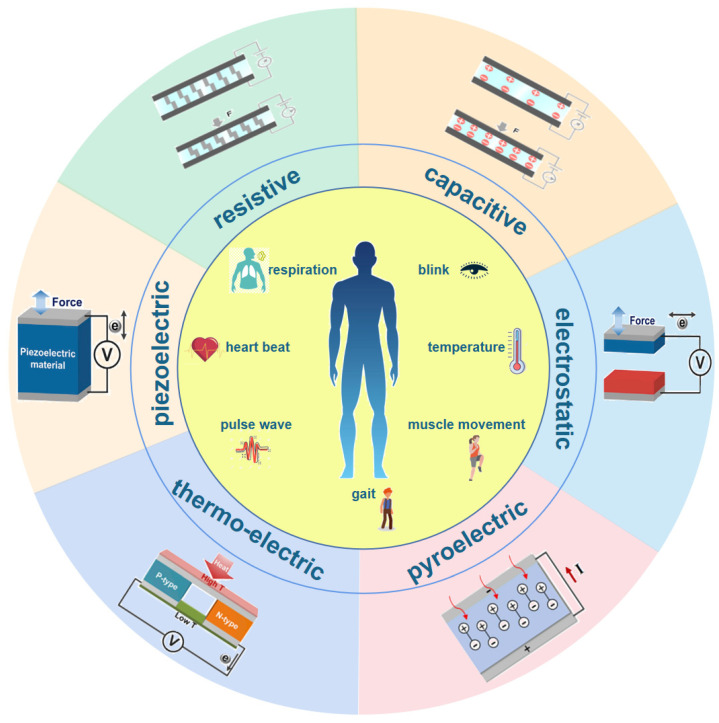
The functionalities of wearable multi-functional sensors and mechanisms, which are resistive, capacitive, triboelectric, piezoelectric, thermo-electric and pyroelectric. Reproduced with permission from Wiley (2019) [11]. Reproduced with permission from Elsevier (2021) [12]. Reproduced with permission from Elsevier (2017) [13].

The fourth industrial revolution, driven by technologies such as the Internet of Things, big data, robots and artificial intelligence, is sweeping the world with unprecedented momentum. Additionally, wearable systems are core parts of the Internet of Things and artificial intelligence. In recent years, wearable systems for human health have attracted broad attention, and many commercial wearable products detecting human body signals continue to appear, such as the Apple Watch, MI band and so on. However, these products cannot attach to human skin tightly, so tiny physiological parameters cannot be perceived. Therefore, flexible, stretchable and multi-functional wearable systems are urgently needed. The front-end components of the system involve all kinds of sensors that detect physiological signals and human movements, such as body temperature [1,2], heart rate [3,4], blood glucose [5,6], blinking [7,8], acceleration [9,10], etc. According to the working mechanism, these sensors can be divided into resistive sensors, capacitive sensors, triboelectric sensors, piezoelectric sensors, thermo-electric sensors, pyroelectric sensors and others fabricated by hybrid mechanisms, as shown in Figure 1 [11,12,13]. 

As important tools to monitor human health, these wearable sensors need to be able to fit tightly to human skin, and there are three commonly used strategies to achieve flexibility and stretchability. Firstly, modifying the side chains of the polymer appropriately can promote geometric bending and stretching. Secondly, fabricating functional materials through thin film mechanics could enhance the flexibility [14]. Another strategy is using natural soft materials, such as cotton [15], silk [16] and wool [17], as the substrate of the textile-based sensors. Apart from the characteristic of being flexible, wearable sensors should have a wide sensing range, from subtle signals (pulse, the mode of respiration [18,19] and the heart rate [20]) to vigorous motions (stretching [21,22], bending [23] and running [24]). Sensitivity is also an important index of wearable sensors, so considerable novel structures and fabrication processes are being developed to achieve high sensitivity.

Conventional wearable sensors need an external power to detect human body signals, which limits the miniaturization of the sensors and will cause environmental pollution. The invention of triboelectric sensors, piezoelectric sensors, thermo-electric sensors and pyroelectric sensors solved this problem by forming a self-powered active sensor. Sensors with hybrid mechanisms could perceive more than one external stimulus, and have great potential for multi-function intellectualization and miniaturization.

This article intends to provide an overview on wearable multi-functional sensing technology for human healthcare, concentrating on the working principles of resistance, capacitance, triboelectricity, piezoelectricity, thermo-electricity, pyroelectricity and hybrid mechanisms. Conclusions and future outlooks in the field of wearable multi-functional sensing technology for human healthcare are discussed in the final section.

## 2. Single Mechanisms

### 2.1. Resistive Principle

**Figure 2:**
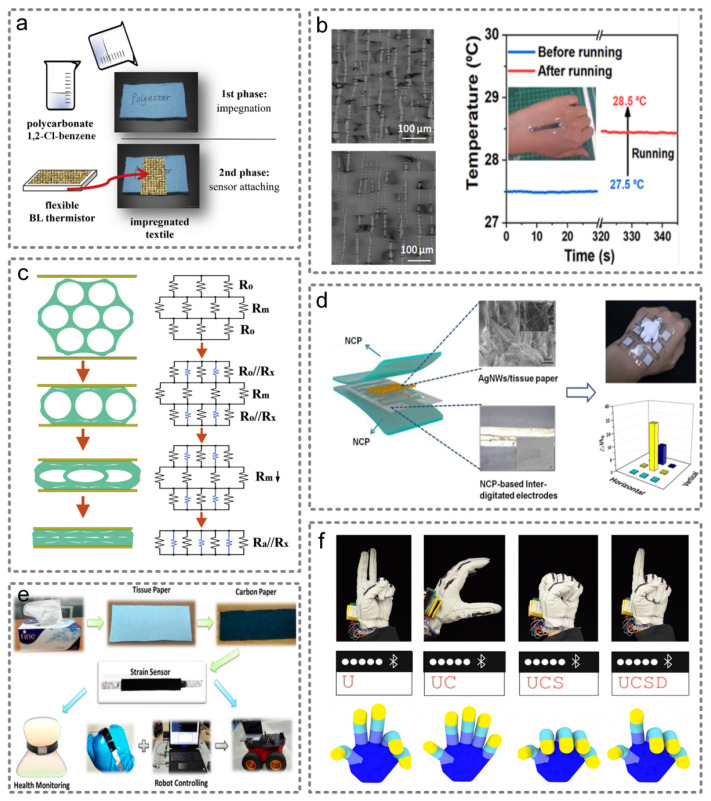
Resistive wearable sensors. (**a**) The impregnation procedure to fabricate a thermistor into a textile. Reproduced with permission from Elsevier (2017) [29]. (**b**) The optical microscope images of different cracks within PEDOT:PSS−PDMS sensors, which could improve the temperature sensitivity and the application of health detection. Reproduced with permission from Elsevier (2020) [30]. (**c**) Schematic illustrations of a network structure and its equivalent circuit diagram. Reproduced with permission from Elsevier (2017) [32]. (**d**) The structure of the piezoresistive senser based on tissue paper coated with silver nanowires (AgNWs). Reproduced with permission from the American Chemical Society (2019) [36]. (**e**) A highly flexible resistive-type strain sensor composed of carbon paper (CP) and polydimethylsiloxane (PDMS) elastomer. Reproduced with permission from the American Chemical Society (2016) [38]. (**f**) The smart glove that can translate American Sign Language into text. Reproduced with permission from Public Library Science (2017) [39].

Resistive sensors can be divided into thermistor sensors, piezoresistive sensors, resistive strain sensors and so on. The principle of thermistors is that the resistivity of materials varies with the environmental temperature. Additionally, numerous materials could be used as the sensing material, such as metals [25], inorganic semiconductors [26], organic polymers [27] and ionic liquids [28]. Among them, organic molecular conductors have especially excellent qualities. On the one hand, they can give electronic devices special strain, pressure and temperature sensing performance that cannot be achieved by metal-based structures [29]. On the other hand, organic molecular conductors can form a thin film through deposition, thus directly affecting the performance of material sensing. Additionally, Lebedev et al. conducted a study, as shown in Figure 2a, that processed a thermistor by combining polycarbonate/α’-(BEDT-TTF)_2_I_x_Br_3-x_ with a polyester textile, which can be directly used in a biomedical and environmental monitor with an accuracy of 0.005 °C [29]. As a wearable temperature sensor, it requires not only high precision, but also high sensitivity and linearity. Yu et al. provided a solution to achieve those indicators [30]. By coating the PDMS with PEDOT:PSS, when the PDMS substrate expands with increasing temperatures, the conductive sensor layer tends to generate microcracks, which significantly improves the temperature sensitivity to 0.042 °C^−1^. The optical microscope images of different cracks within PEDOT:PSS−PDMS sensors and the application are shown in Figure 2b.

Piezoresistive sensors are devices based on the piezoresistive effect. In the past few decades, researchers have explored a variety of novel materials and structures, and have designed flexible wearable piezoresistive pressure sensors. These kind of sensors are mainly based on 3D monolithic conductive sponges [31]. Under pressure, the temporary contact area/points of the porous conductive sponge increase, forming more conductive paths, resulting in a lower resistance. In addition, the number of temporary contacts is determined by compressive strain. Therefore, piezoresistive pressure sensors can detect both transient and static deformation. The structure of the 3D conductive sponge and its equivalent circuit diagram is shown in Figure 2c [32]. Additionally, these piezoresistive sensors also have the advantages of simple fabrication, a wide detection range, a fast response speed and so on. Three-dimensional conductive sponges can be divided into four types according to their structure and composition: (1) neat conductive sponges, (2) composite conductive sponges, (3) conductive sponges impregnated with elastomer and (4) conductive material-coated sponge. Neat conductive sponges are 3D porous sponge structures assembled solely from conductive materials. Carbon nanotubes (CNTs) and graphene are commonly used materials [31]. Composite conductive sponges are sponges consisting of a blend of polymer and conductive materials. For example, Xu et al. reported a 3D conductive sponge nanostructure assembled from polyacrylonitrile (PAN) and polyimide (PI) [33]. Conductive sponges impregnated with elastomer are sponges prepared by permeating elastic polymers into neat conductive sponges, which could enhance their stability and compressibility. Additionally, Wang et al. developed a piezoresistive sensor by impregnating non-woven materials with nano-silver-conductive ink [34]. Conductive material-coated sponges are sponges formed by coating conductive material on a non-conductive polymer sponge via sputtering, dip coating, in situ polymerization and other methods. For example, Kannichankandy et al. made the ultra-low-cost and biodegradable piezoresistive pressure sensors by coating polyaniline (PANI) on cellulose paper [35]. Additionally, Gao et al. presented a pressure sensor based on a tissue paper coated with silver nanowires (AgNWs) as a sensing material, as shown in Figure 2d. [36] It is worth noticing that Shi et al. developed a new method to fabricate piezoelectric pressure sensors by electroplating Ni film on PI (polyimide) matrix. When pressing the sensor, microcracks will occur on the tip of the Ni layer, resulting in the change of resistance [37]. 

Another kind of resistive sensor is the strain resistive sensor, which works by converting mechanical deformations into resistance value changes. It is usually composed of a sensitive element and a support structure. Polydimethylsiloxane (PDMS) is a commonly used substrate material, which has stable chemical properties, flexibility, biocompatibility, transparency and good thermal stability [40]. For example, Yamada et al. fabricated a PDMS-based strain sensor using an aligned, single-walled carbon nanotube (CNT) film, which is capable of measuring up to 280% strain [41]. Different motion signals can be detected when sensors are located in different parts of the body. Additionally, Li et al. fabricated a flexible resistive strain sensor composed of carbon paper (CP) and a polydimethylsiloxane (PDMS) elastomer, as shown in Figure 2e [38]. O’Connor et al. presented a smart glove, whose key parts are resistive strain sensors. This glove is capable of translating American Sign Language into text on the screen, which suggests a new way for humans to interface with the virtual environment, as shown in Figure 2f [39]. 

### 2.2. Capacitive Principle

The mechanism of capacitive sensors is based on the formula:

$$C=\frac{\varepsilon_{0}\varepsilon_{r}S}{d}$$

where ε_0_ is the permittivity of free space, ε_r_ is the relative permittivity of the dielectric material, S is the overlapping area, and d is the distance between the two electrodes. The thickness of the dielectric layer and the distance between the electrodes will change under the external force, meaning that the capacitance is changed. The value of the external pressure can be generated by detecting the change of capacitance.

Traditional capacitor structures are usually made of hard materials, which makes them difficult to attach to the skin to obtain accurate physiological signals. Therefore, the capacitive sensors for human health should be flexible, stretchable, light, tiny and highly sensitive. In order to achieve the requirements of flexibility and ductility, new composite materials are usually selected for the electrodes and the dielectric layer of capacitors.

**Figure 3:**
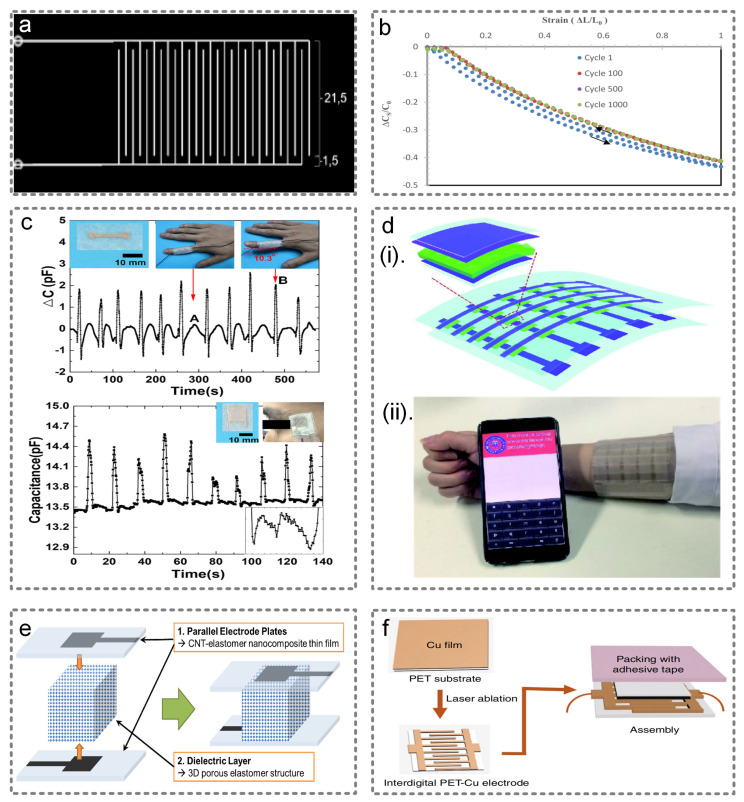
Capacitive wearable sensors. (**a**) A capacitive sensor using barium titanate–EcoflexTM 00-30 composite as the substrate and CB/EcoflexTM 00-30 composite conductive ink as the interdigital electrodes. (**b**) The relative capacitance change strained up to 100% after 1, 100, 500 and 1000 stretch/relax cycles. Reproduced with permission from MDPI (2018) [42]. (**c**) The capacitance profile of finger motion and eye blinking. Reproduced with permission from MDPI (2019) [43]. (**d**) The structure and its application as a flexible keyboard. Reproduced with permission from Springer Nature (2018) [46]. (**e**) Schematic illustration of the sensor with a foamy dielectric layer [48]. (**f**) The structure of the flexible pressure sensor based on MXene/PVP membrane and interdigital PET-Cu electrodes. Reproduced with permission from Springer Nature (2021) [45].

Cholleti et al. selected a barium titanate–Ecoflex^TM^ 00-30 composite as the capacitor substrate that can withstand up to 100% ductility, and a CB/Ecoflex^TM^ 00-30 composite conductive ink was selected as the material of the electrodes [42]. Then, the IDC structure was printed on the substrate via 3D printing, as shown in Figure 3a. The relative change in capacitance when strained up to 100% after 1, 100, 500 and 1000 stretch/relax cycles is shown in Figure 3b. As the capacitor is stretched or compressed, the space between the electrodes changes, and the capacitor value changes accordingly. Xie et al. used napkins as the substrate to self-assemble silver nanowires on PDMS patterned napkins to instill flexibility in the electrode plates [43]. The capacitance profiles of finger motion and eye blinking are shown in Figure 3c. The capacitive sensor can detect a variety of physiological signals, such as finger movement, blinking, pulse and so on. Additionally, recent works focus the dielectric elastomers using elastomer insulating layers (e.g., silicone rubber) sandwiched between two layers of deformable electrode materials (e.g., carbon black loaded silicone rubber) to obtain stretchable capacitors to sense different body positions [44].

To achieve good sensing accuracy, the following two strategies are commonly used. The first one is to introduce a microstructure onto the electrode or dielectric layer to obtain a rough plane [45]. For example, Shi et al. graphically achieved higher sensitivity and stability in the mixed media of silver nanowires and PDMS with a microstructure. The structure and its application as a flexible keyboard are shown in Figure 3d [46]. Additionally, Bai et al. reported on intrafillable microstructures that can not only improve the sensitivity, but also broaden the pressure range [47]. The second method is the use of a porous or foamy dielectric layer structure, which can add air to the dielectric layer so as to adjust the dielectric constant. Here, even if a small amount of pressure is given, an obvious deformation will be produced, so that the output of the change in the capacitance value is larger and the sensitivity is improved. As shown in Figure 3e, the capacitor structure designed by Kwon et al. has an ultra-high sensitivity of 0.601 kPa^−1^ and a wide working range of 0~130 kPa, which can be used for the measurement of human pulse signals [48]. Apart from the two methods mentioned above, the research of Qin et al. provided a new strategy for improving the sensitivity of capacitive sensors [45]. By increasing the number of interdigital electrodes, and through the reasonable selection of the dielectric layer material MXene/polyvinylpyrrolidone (PVP), the sensitivity of a capacitor has been improved, and its structure is shown in Figure 3f. 

### 2.3. Triboelectric Principle

Triboelectric sensors are based on triboelectric nanogenerators (TENG). The friction layers of TENG make contact with each other under external forces, creating inductive charges on the surface of the friction layer due to the difference in their electron affinities. When the external force disappears, an internal potential is formed, causing the current to flow from one electrode to another. Additionally, the sensing function is realized by analyzing the current signals [49]. 

Since the invention of triboelectric nanogenerators in 2012, the sensors that use electrostatic induction to detect human motion patterns came along correspondingly [50]. The triboelectric nanogenerators could convert the collected signals of human movements into electrical signals, so the sensor can work without an external power source. The materials commonly used as friction layers are PTFE, PDMS, PI, nylon [51] copper and silver, which easily lose or gain electrons. Recently, a variety of new functional materials have been used in triboelectric sensors for human health. For example, hydrogel is widely used for the electrodes in triboelectric sensors, which can detect the arbitrary movements of human body, such as distortion, stretch and so on [52]. Additionally, Zou et al. have used ionic liquids and fabricated a bionic structure sensor that can monitor multiple positions of the human body underwater [53]. Significantly, textile-based wearable sensors are the new trend for detecting physiological signals [54,55].

**Figure 4:**
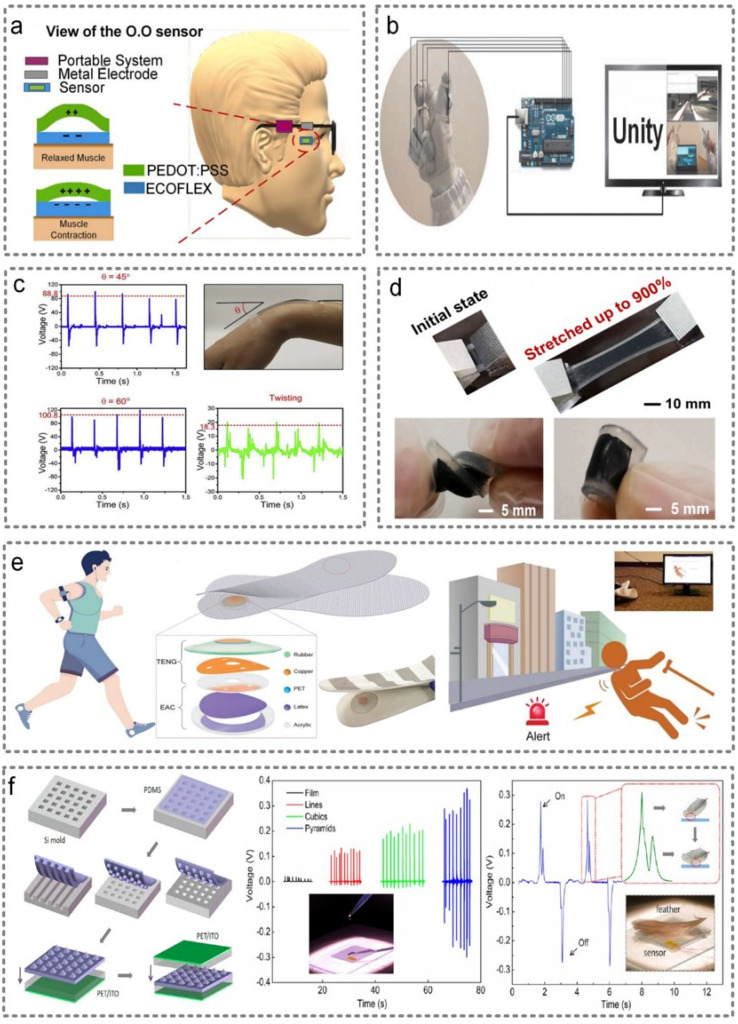
Triboelectric wearable sensors. (**a**) A novel structure named Non-Attached Electrode-Dielectric Triboelectric Sensor (NEDTS) that can detect eye motion. Reproduced with permission from Elsevier (2020) [56]. (**b**) The minimalistic design of a glove made up of several triboelectric textile sensors. Reproduced with permission from Wiley (2020) [57]. (**c**) The printed silk fibroin-based triboelectric nanogenerator that could realize the recognition of joint motion. Reproduced with permission from Elsevier (2019) [58]. (**d**) The mechanical characterization of the super-stretchable TENG. Reproduced with permission from Elsevier (2019) [59]. (**e**) Smart insoles assembled into the shoes to serve as a self-powered gait monitoring system and a warning system for falling down. Reproduced with permission from Wiley (2018) [60]. (**f**) Three kinds of polymer pattern arrays (line, cube and pyramid) in the electrification layer that could improve their sensitivity. Reproduced with permission from the American Chemical Society (2012) [63].

Triboelectric sensors are widely used due to their advantages of requiring no external power supply, their simple principle and the wide selection of materials. The traditional triboelectric nanogenerators mainly have four modes, which are single-electrode mode, relative-sliding mode, contact-separation mode and free-standing mode [49]. In addition to these four structures, Anaya et al. presented a novel structure named Non-Attached Electrode-Dielectric Triboelectric Sensor (NEDTS), which eliminated the assembly steps of bonding nanowires on dielectric polymers or depositing conductive materials [56]. Additionally, sensors of this structure could be used to detect eye motion, as shown in Figure 4a, while the contraction or relaxation of the eye muscles can make the PEDOT:PSS film and ECOFLEX™ touch each other, inducing charges in the metal. Then, the portable system processes the electric signals to sense eye motion. Apart from being applied to detect eye motion, Wen et al. combine several triboelectric textile sensors and advanced machine learning, and achieved highly accurate virtual/augmented reality (VR/AR) control with a glove that has a minimalistic design, as shown in Figure 4b [57]. A similar work conducted by Wen et al. could realize the recognition of joint motion with the help of a printed silk fibroin-based triboelectric nanogenerator, as shown in Figure 4c [58]. It is worth mentioning that Deng et al. developed a carbon nanotube (CNT)–silicone rubber liquid composite, and fabricated a super-stretchable triboelectric nanogenerator (SS-TENG) that can achieve 900% stretchable deformation based on this material. The mechanical characterization of the super-stretchable TENG (SS-TENG) is shown in Figure 4d [59]. Additionally, a self-powered wearable keyboard was achieved based on SS-TENG arrays. Additionally, Lin et al. developed a TENG-based smart insole for real-time monitoring of human gait, which can accurately monitor gait patterns, including jumping, footsteps, walking and running, by analyzing TENG signal differences, as shown in Figure 4e [60]. Therefore, this sensor can be used as a fall warning system for the elderly or patients in the field of health monitoring. In the same year, Wang et al. created a smart mask that can drive a triboelectric nanogenerator and generate electrical signals through breathing, realizing real-time monitoring of breathing patterns [61]. Additionally, Peng et al. reported an all-nanofiber TENG-based biodegradable e-skin, which is able to detect physiological signals (pulse, vocalization and respiration) and joint movements (elbow, knee and ankle) in a self-powered manner [62].

In order to improve the sensitivity of triboelectric sensors, microstructures are often introduced into the electrification layer. For example, Fan et al. fabricated three kinds of polymer pattern arrays (line, cube and pyramid) [63]. In this way, the output voltage could reach 18 V when the current density was only 0.13 μA/cm^2^. Therefore, this device can be used to sense a droplet of water and the falling of a feather, as shown in Figure 4f. 

### 2.4. Piezoelectric Principle

Piezoelectric sensors are fabricated based on piezoelectric material. When piezoelectric materials are subjected to an external force, an internal polarization phenomenon will occur, and the positive and negative charges will appear on the two surfaces of the piezoelectric material, forming a piezoelectric potential. When the external force is withdrawn, the polarization phenomenon will disappear. By detecting the change of the electrical signal, it can be used to realize pressure sensing.

The most commonly used piezoelectric materials are PZT, BaTiO_3_, PbTiO_3_, ZnO, PVDF, polypropylene (PP) and so on [64]. However, when the traditional piezoelectric materials are subjected to pressure, cracks will appear, which hinders the performance of piezoelectric sensors. Yang et al. introduced polydopamine (PDA) to barium titanate (BaTiO_3_,BTO) as a surface modification agent, then blended them with a poly(vinylidene fluoride) (PVDF) matrix to form PDA@BTO/PVDF composites [65]. In this way, hole defects and cracks are reduced.

**Figure 5:**
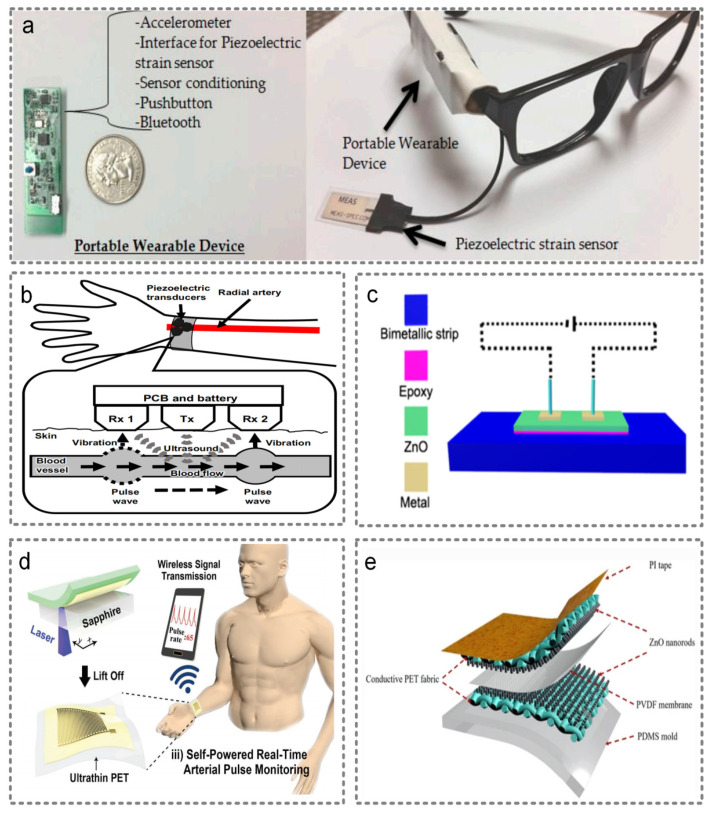
Piezoelectric wearable sensors. (**a**) A novel wearable piezoelectric sensor placed on the temporalis muscle to detect food intake. Reproduced with permission from MDPI (2016) [69]. (**b**) The structure of the multiple piezoelectric transducers that can measure blood flow velocity and pulse simultaneously. Reproduced with permission from Springer Nature (2018) [70]. (**c**) The structure of the piezoelectric sensor based on ZnO nanowire (NW) film that is able to sense temperature. Reproduced with permission from American Chemical Society (2014) [71]. (**d**) An ultra-thin flexible self-powered piezoelectric pulse sensor based on PZT film. Reproduced with permission from Wiley (2017) [73]. (**e**) A textile piezoelectric pressure sensor (T-PEPS). Reproduced with permission from Springer Nature (2021) [76].

Sensors based on piezoelectric effects are widely used to detect all kinds of signals from the human body, such as those applied to the nose to detect respiratory patterns, those applied to the wrist to detect pulse signals or record fingers movements [66], and those applied to the chest to obtain the heart rate signals [67]. For example, Manjunatha et al. presented a nasal sensor based on a piezoelectric polyvinylidene fluoride (PVDF) thin film to monitor respiration patterns [68]. The air exhaled from the nostrils hits the cantilever beam, creating a dynamic voltage signal based on piezoelectric effects. Then, according to the recorded voltage, we are able to analyze the respiration pattern, which is of great significance to monitor sleep apnea syndrome and lung health. Farooq et al. presented a novel wearable piezoelectric sensor placed on the temporalis muscle to detect food intake [69]. By taping the piezoelectric film sensor on the temporalis muscle, they were able to integrate the accelerometer with another signal processing unit in a PCB board. Finally, one can achieve a portable wearable device by using a simple glass structure, as shown in Figure 5a. Okano et al. proposed a method that can measure blood flow velocity and pulse simultaneously [70]. The multiple piezoelectric transducers can detect a pulse on multiple positions on the wrist, and simultaneously send ultrasound waves toward the body surface. By detecting the frequency of reflected ultrasound waves, the blood flow velocity is measured. The structure of this device is shown in Figure 5b. Apart from sensing pressure, piezoelectric sensors are also able to sense temperature. For example, Xue et al. fabricated ZnO nanowire (NW) film utilizing the wet chemical deposition method, and transferred it to a flexible substrate. The structure is shown in Figure 5c. When the temperature fluctuates, the deformation of the film will occur. By measuring the induced potential of this piezoelectric material, the parameter of temperature is detected [71]. 

It is worth noticing that piezoelectric sensors can also achieve self-powered functionality. For example, Liu et al. fabricated a nanogenerator and used it as a flexible active respiratory sensor [72]. Park et al. demonstrated an ultra-thin, flexible, self-powered piezoelectric pulse sensor based on PZT film, which can be in close contact with the skin to achieve high sensitivity [73]. Finally, the pulse signal is transmitted to mobile phone via Bluetooth, and a complete set of pulse detection systems is realized as shown in Figure 5d. Importantly, the state-of-the-art electrospun nanofiber technology has become one of the main ways to effectively prepare nanofiber materials due to its advantages of being a simple manufacturing device, its low spinning cost, the wide variety of spinnable materials and the controllable process. Additionally, piezoelectric energy harvesters based on electrospun nanofiber technology are a cutting-edge research trend. Roy et al. fabricated a piezoelectric nanogenerator based on CdS/rGO nanocomposites doped electrospun PVDF nanofiber, which has the advantages of being light weight, flexible and biocompatible [74]. Ponnan et al. proposed a piezoelectric nanogenerator using an n-PVDF and PVDF−MNC nanofiber system that dramatically enhanced piezoelectric characteristics [75].

However, so far, many high-performance flexible piezoelectric nanogenerator are made of lead-containing materials, such as Pb(Zr,Ti)O_3_, PZT and so on, which are poisonous and not suitable for biological applications. Apart from lead-based materials, other organic and inorganic materials are also non-biocompatible and non-biodegradable. To solve this severe problem, a lot of research has been conducted to explore biocompatible and biodegradable materials. Tao et al. demonstrated a degradable piezoelectric energy harvester based on a free-standing polylactic acid film with embedded diphenylalanine microrods arrays [77]. Interestingly, some natural materials, such as collagen-based biowaste fish skin (FSK) [78] and cellulose-based onion skin [79], are also considered to be effective biodegradable piezoelectric materials.

In order to promote the comfort level of wearing sensors, Tan et al. fabricated a textile piezoelectric pressure sensor (T-PEPS) and simultaneously harvested mechanical energy [76]. The structure is shown in Figure 5e. The T-PEPS is constructed with three layers, consisting of a polyvinylidene fluoride (PVDF) membrane, and top and bottom layers made of conductive rGO polyester (PET) fabrics with self-orientation ZnO nanorods, which has high sensitivity and wide pressure range.

### 2.5. Thermo-Electric Principle

Thermo-electricity means the direct conversion of heat into electric energy, or vice versa. If a closed loop is formed by joining the ends of two strips of dissimilar metals, and the two junctions of the metals are at different temperatures, a voltage arises that is proportional to the temperature difference between the junctions. If a current passes through a thermocouple, the temperature of one junction increases and that of the other decreases. The rate of heat transfer is proportional to the current. A lot of materials, such as Bi_2_Te_3_, have been discovered lately [80]. Additionally, the flexible thermo-electric nanogenerator, based on thermo-electric materials, is often used in thermo-electric sensors.

Thermo-energy is the most acquirable source in nature, and the human skin can be seen as a source of variable thermal resistance. Therefore, thermo-electric sensors could be applied to a wide range of fields. Commonly used thermo-electric materials are Bi_2_Te_3_-based inorganic materials and their alloys, and some novel thermo-electric materials have been explored in recent years, such as a nanocomposite developed by Madavali et al., the spark plasma sintered Na-doped PbTe:SrTe, the p-type PEDOT doped with PSS and so on [81].

**Figure 6:**
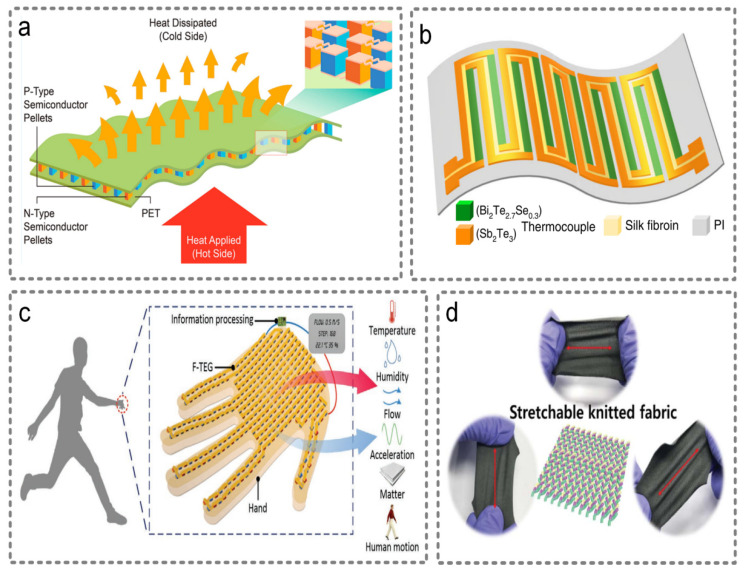
Thermo-electric wearable sensors. (**a**) The structure of a thermo-electric nanogenerator (FTEG), which is constructed with 126 thermo-electric legs laid in an array to sense temperature. Reproduced with permission from the American Chemical Society (2019) [82]. (**b**) The structure of a double-chain thermo-electric generator. Reproduced with permission from Springer Nature (2020) [84]. (**c**) The e-skin that can sense temperature, humidity, acceleration, motion and so on. Reproduced with permission from Wiley (2020) [85]. (**d**) The textile-based sensor processed with commercial thermo-electric inks. Reproduced with permission from the Royal Society of Chemistry (2018) [86].

Figure 6a shows a flexible thermo-electric nanogenerator (FTEG), which is constructed with 126 thermo-electric legs laid in an array [82]. Using a zone-melting and nanofabrication process, this FTEG based on Bi_2_Te_3_ can reach the temperature resolution of 0.5 K, which has a bright prospect in the wearable physical sensor field. Apart from sensing temperature, thermo-electric sensors are also able to sense external pressure. Oh et al. proposed a thermo-electric pressure sensor in a coaxial strut structure with a fractured microstructure, and it could attain high sensitivity [83]. Figure 6b shows a double-chain thermo-electric generator [84]. The two chains can not only harvest thermal energy, but also work as the sensing electrodes. Additionally, silk fibroin, which covers the gap of the two chains, could perceive the existence of liquid-state water in the air. Furthermore, the e-skin, driven by human body heat, could realize the function of sensing temperature, humidity, acceleration, motion and so on [85]. Figure 6c shows the e-skin fabricated by Yuan et al. The core part of the e-skin is a flexible thermo-electric nanogenerator, which is made of bismuth antimony telluride grains assembled on a flexible polyimide film. By testing the output voltage, the external stimuli could be sensed.

Some textiles have the advantages of stretchability, comfort, thinness, flexibility and could be manufactured easily. Jung et al. provided a solution to achieve better comfortability and easy processibility with thermo-electric sensors [86]. As shown in Figure 6d, it is a textile-based sensor processed with commercial thermo-electric inks, which has a temperature-sensing ability, depending on the stretching directions.

Although the wearable thermo-electric sensors have achieved tremendous progress in recent years, there are still some remaining problems that need to be tackled. For example, the human thermoregulatory models need to be accurately simulated, and the flexible heat sinks also need to be considered [87].

### 2.6. Pyroelectric Principle

Unlike the thermo-electric materials, pyroelectric materials are usually known to be insulating, and they will present a spontaneous polarization when there is no external electric field. Additionally, when temperature fluctuates, the polarization of pyroelectric materials changes, leading to a corresponding voltage. Pyroelectric materials have been widely explored, such as polymers (polyvinylidene fluoride; PVDF), triglycine sulfate (TGS), bulk perovskite ceramics and so on [88]. Among them, PVDF is widely used because it can be fabricated into thin films and has good mechanical robustness. 

**Figure 7:**
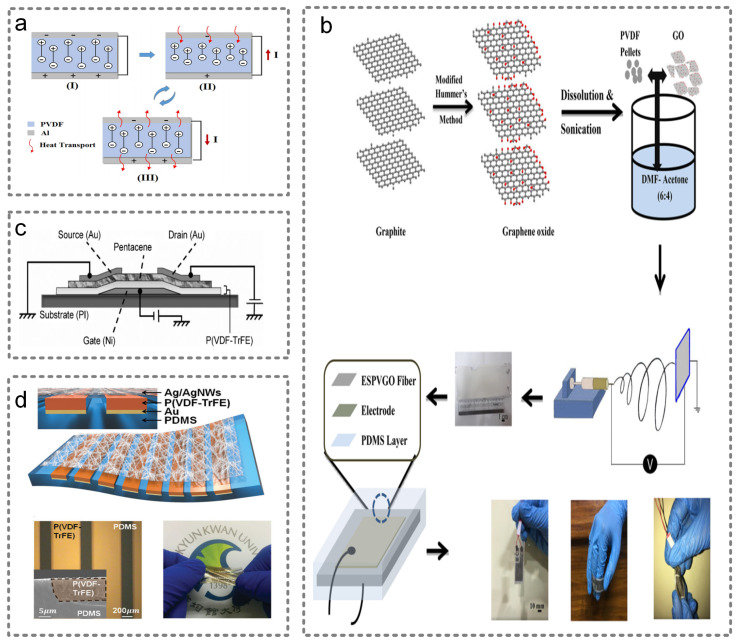
Pyroelectric wearable sensors. (**a**) The working mechanism of the pyroelectric nanogenerator. Reproduced with permission from Elsevier (2017) [13]. (**b**) The fabrication route of an ultra-sensitive temperature sensor based on PVDF/graphene oxide (GO) nanofibers. Reproduced with permission from the American Chemical Society (2019) [89]. (**c**) The organic field-effect transistor with P(VDF-TrFE) layer. Reproduced with permission from Wiley (2009) [90]. (**d**) The structure, OM image and the photo image of the stretchable pyroelectric device. Reproduced with permission from Wiley (2015) [91].

Xue et al. fabricated a pyroelectric nanogenerator based on PVDF, with the maximal power to be 8.31 μW [13]. The working mechanism of the pyroelectric nanogenerator is shown in Figure 7a. By analyzing the output signal, it can be used as a human breathing and temperature sensor. Roy et al. conducted a similar study, and presented an ultra-sensitive temperature sensor based on PVDF/graphene oxide (GO) nanofibers, which can sense the temperature change during breathing, and it can also harvest thermal energy by working as a nanogenerator [89]. The fabrication route is illustrated in Figure 7b. Apart from PVDF, Tien et al. selected the P(VDF-TrFE) material, and integrated it into an organic field-effect transistor as the insulation layer, as shown in Figure 7c [90]. Additionally, the temperature response of this device was mainly affected by the P(VDF-TrFE) layer. Lee et al. fabricated a stretchable pyroelectric device based on micropatterned P(VDF–TrFE) and PVDF. The structure of this device, its OM image and the photo image are shown in Figure 7d. Additionally, their work suggested that the micropatterned structure is beneficial to stretchability and durability [91].

To increase the temperature sensing range, Cosseddu et al. proposed a novel structure of a temperature sensor by coupling a field-effect transistor with a pyroelectric element [92]. The pyroelectric principle can also be applied to the gesture recognition field. Gong et al. developed signal processing software based on a pyroelectric sensor [93]. This system can recognize six gestures, including a triangle, rectangle, check mark, circle, question mark, and finger rub, which offers great potential for human wearable devices.

## 3. Hybrid Mechanisms

### 3.1. Two-Principle Integration

The wearable sensors based on a single mechanism are unable to detect multiple parameters and could not meet the requirement of multi-functional sensing. Therefore, it is indispensable to develop sensors based on hybrid mechanisms. The following section will introduce the recent progress on wearable sensors based on two-principle integration.

**Figure 8:**
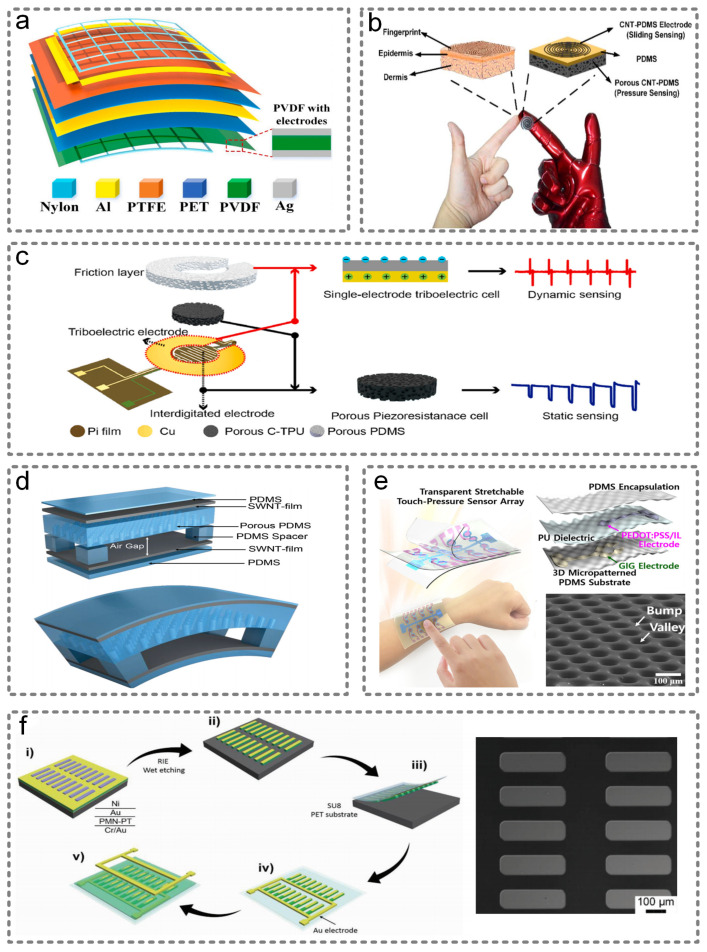
Two-principle integrated wearable sensors. (**a**) A self-powered sensor based on the integration of the triboelectric and piezoelectric mechanisms, which can detect the finger sliding trace and vertical force. Reproduced with permission from Elsevier (2021) [94]. (**b**) A comparison diagram of fingertip and e-skin. Reproduced with permission from Elsevier (2017) [96]. (**c**) A triboelectric active sensing unit based on porous polydimethylsiloxane (PDMS) and a piezoresistive sensing unit based on a conductive carbon black (CB)/thermoplastic polyurethane (TPU) composite that are vertically integrated. Reproduced with permission from Elsevier (2020) [97]. (**d**) The structure of e-skin that can not only detect the usual pressure, strain and bending, but also perceive lateral strain. Reproduced with permission from Wiley (2014) [100]. (**e**) The transparent flexible sensor that can detect touch and pressure through capacitive and piezoresistive effect. Reproduced with permission from Springer Nature (2019) [101]. (**f**) Schematic illustration of the fabrication process for a flexible PMN-PT ribbon-based generator and sensor on plastic substrates, and its SEM image. Reproduced with permission from Wiley (2017) [103].

Tang et al. presented a self-powered sensor based on the integration of the triboelectric and piezoelectric mechanisms [94]. The triboelectric unit with four electrodes can detect the finger sliding trace in x-y plane, while the piezoelectric unit detect the finger force in the z direction. The specific structure is shown in Figure 8a, which has great potential to be applied in diverse human–machine interactions, VR/AR, wearable electronics, etc.

In addition to the triboelectric and piezoelectric integration mechanism, Zhang et al. reported an active sensor based on the integration of resistive and triboelectric mechanisms that can detect both the static gestures of the human body and prosthesis motion [95]. When detecting static gestures, the resistance will increase as the contact area of the conductive layer is reduced. Additionally, when detecting dynamic motions, the sensor will work as a triboelectric nanogenerator. Therefore, it could work as a smart skin that is able to track fingers, elbows, knees and other joint motions. A similar work conducted by Chen et al. can also realize the tracking of fingers [96]. The device includes double spiral carbon nanotube-poly-dimethylsiloxane (CNT-PDMS) electrodes, substrate and porous CNT-PDMS, which correspond to fingerprint, epidermis and dermis. Additionally, the comparison diagram of the fingertip and e-skin sensors is shown in Figure 8b. When the fingertip slides along the surface, it will create an alternating voltage due to the triboelectrification effect, so the sliding sensing is realized. Additionally, the pressure sensing will be achieved by detecting the change of the resistance when pressing on it. Meng et al. integrated a triboelectric active sensing unit based on porous polydimethylsiloxane (PDMS) and a piezoresistive sensing unit based on a conductive carbon black (CB)/thermoplastic polyurethane (TPU) composite integrated vertically, as shown in Figure 8c [97]. The hybrid sensor could realize the identification of different boxing punches. 

Another kind of hybrid sensor is based on the integration of piezoelectric and resistive mechanisms. Park et al. proposed a smart skin made of a PVDF and reduced graphene oxide (rGO) microdome structure [98]. Due to the piezoelectric and piezoresistive nature of the PVDF-rGO composites, it could perceive static and dynamic pressure, as well as the temperature.

As for the integration of capacitive and resistive mechanisms, abundant research has been conducted in recent years. Chen et al. fabricated a tactile force sensor based on the TSMC standard CMOS process [99]. A capacitive sensing membrane could detect the force on the vertical direction, and piezoresistive bridges could detect the sheer force. Park et al. demonstrated, for the first time, an e-skin that can not only detect the usual pressure, strain and bending, but could also perceive the lateral strain [100]. At the same time, this device can also harvest the energy caused by external stimuli and power the e-skin itself or other wearable sensors. The architecture of this sensor is shown in Figure 8d. Additionally, Hwang et al. reported a transparent flexible sensor that can detect touch and pressure through the capacitive and piezoresistive effects [101]. The capacitive sandwich structure is shown in Figure 8e. By measuring the current caused by the change in the capacitance, the external touch can be detected. By measuring the decrease in the current caused by the change in the piezoresistance of the upper electrode, the pressure can be detected. In addition, it could be integrated into a stretchable touch panel, so it has great potential for use in wearable devices.

With regard to the integration of piezoelectric and thermo-electric mechanisms, Zhu et al. made use of organic piezoelectric poly(vinylidene fluoride) and thermo-electric polyaniline (PANI)-based composite films, and fabricated a flexible sensor that can simultaneously perceive tactile stimuli and temperature without interference [102]. Additionally, this dual-functional sensor could sense elbow bending, pulse and pronunciation.

On account of all pyroelectrics being piezoelectric materials, several multi-functional sensors combining pyroelectric and piezoelectric effects have been developed. For example, Figure 8f shows the 0.7PMN-0.3PT ribbon-based hybrid sensor on a flexible polymer substrate fabricated by Chen et al., which could sense acoustic signals and the temperature [103].

Therefore, as a novel type of sensor, hybrid sensors have great potential in the multi-function and miniaturization trend. Additionally, the exploration of the hybrid mechanisms will be a focus in the future.

### 3.2. Three-Principle Integration

**Figure 9:**
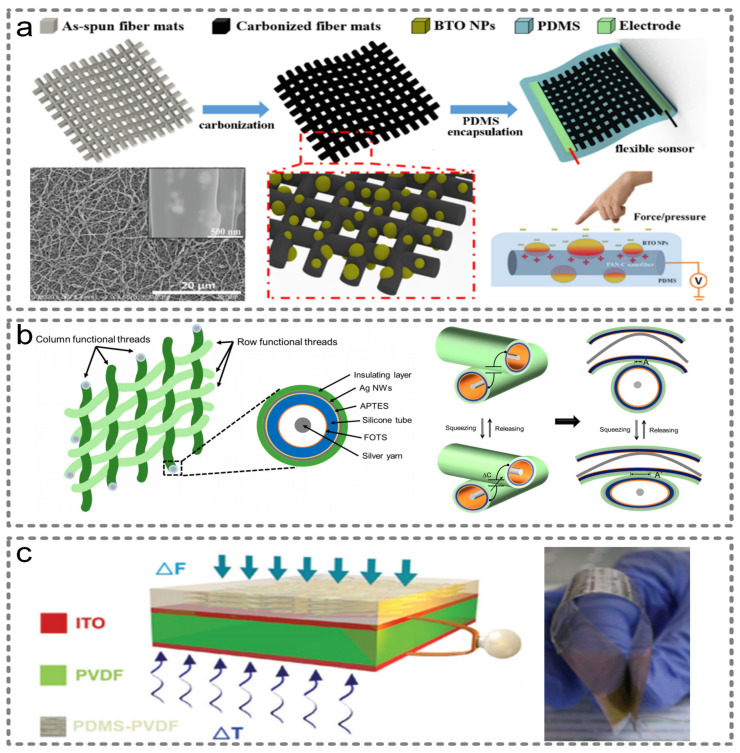
Three-principle integrated wearable sensors. (**a**) A multi-functional sensor made up of carbonized electrospun polyacrylonitrile/barium titanate (PAN-C/BTO) nanofiber film. Reproduced with permission from the American Chemical Society (2018) [104]. (**b**) A power-generating sensor based on the integration of the capacitive, resistive and triboelectric mechanisms. Reproduced with permission from MDPI (2017) [105]. (**c**) A flexible multi-functional sensor combining triboelectric, piezoelectric and pyroelectric effects. Reproduced with permission from Wiley (2016) [106].

To meet the growing demands for highly efficient flexible devices and to achieve more functions in a single wearable sensor, more sensing mechanisms need to be integrated. Zhao et al. reported a multi-functional sensor made up of a carbonized electrospun polyacrylonitrile/barium titanate (PAN-C/BTO) nanofiber film, which is shown in Figure 9a [104]. By combining piezoelectric, piezoresistive and triboelectric effects, this high-end sensor could perceive two parameters (pressure and curvature) independently and simultaneously. The sensitivity is enhanced on account of the mix of barium titanate nanoparticles (BTO NPs), so that multiple human motions, such as finger tapping, swallowing and gait, can be detected.

Kim et al. integrated the capacitive, resistive and triboelectric mechanisms and fabricated a power-generating sensor as shown in Figure 9b [105]. The vertical force can be sensed by measuring the capacitance between the warp and weft functional threads. Additionally, the lateral strain can be detected by measuring the resistance change. In addition, the conductive silver yarn rubs against the inside wall of the tube, so the energy is generated. 

Wang et al. combined triboelectric, piezoelectric and pyroelectric effects, and made a triple-hybrid transparent and flexible nanogenerator [106]. The triboelectric layer is a PVDF nanowires–poly(dimethylsiloxane) (PDMS) composite film, the piezoelectric and pyroelectric layers are made of polarized PVDF film and the electrodes are made of indium tin oxide (ITO), as shown in Figure 9c. Multiple hybrid nanogenerators can collect different types of energy based on various mechanisms, so they can also be seen as multi-functional sensors [107]. 

In summary, the emergence of wearable smart sensors has brought us a new way of life. All kinds of functions depend on the integration and innovation of various mechanisms. Therefore, it is necessary to develop more accurate, miniaturized and integrated sensors to meet the demands of healthcare smart detection. 

## 4. Conclusions and Outlooks

This review focuses on the single physical mechanisms (resistive, capacitive, triboelectric, piezoelectric, thermo-electric, pyroelectric) of wearable sensors and the hybrid mechanisms of multi-functional wearable sensors. In the case of sensing mechanisms, each type has pros and cons, and a trade-off between performances is still necessary. For resistive sensors, there are numerous materials that could be used as the sensing material, such as metals, inorganic semiconductors, organic polymers and ionic liquids. However, an obvious nonlinearity will occur when a large strain is applied. Compared with other types of pressure sensors, capacitive pressure sensors have high accuracy, low power consumption and good transient response [46]. However, the influence of parasitic capacitance will make the output signals unstable and affect the accuracy. Triboelectric sensors with simple structures have obtained great success in the field of wearable electronics, while their output signals are easily influenced by environmental conditions or other parameters, such as humidity. Piezoelectric sensors also have simple structures and are easy to scale down to be integrated into MEMS. However, this type of sensing mechanism is limited by the materials. As for thermo-electric sensors, they usually have high precision and durability, while at the same time, the selection of materials is limited. Pyroelectric sensors have a wide range of operating temperatures, but have a limitation with the temperature fluctuation frequency [108]. Most importantly, for the last four of those sensing technologies (triboelectric, piezoelectric, thermo-electric, pyroelectric), their mechanisms could convert the energy from the ambient environment into electricity while sensing physiological signals to achieve self-powered functionality.

These smart sensors have the characteristics of being flexible, stretchable, bendable, light, durable, body attachable and stable in the long term. The novel materials and structures are also discussed, which are applied to achieve high sensitivity and self-powering abilities. Additionally, the applications for detecting human physiological signals and motion signals are given.

Though wearable sensors have achieved much progress, the real-life applications of wearable sensors in monitoring human health conditions still have challenges. First of all, with the increasing functions of wearable devices, more mechanisms or more sensors need to be integrated. However, the volume of wearable devices is limited, which requires high integration. Second, due to the structure of the human body, how wearable devices can better fit human skins also needs to be explored in the future. It is still a big challenge to achieve high resolution, high sensitivity, fast response, low-cost manufacturing and complex signal detection for flexible wearable electronic sensors. As for the future trend in the field of wearable sensors, precision medicine is an emerging medical field, and wearable sensors play a significant role in this field. At present, all kinds of physiological signals and life habit detection has been realized by utilizing wearable sensors, which dramatically improves the quality of medical diagnosis. Secondly, with the rapid development of the materials, science and the processing technology, novel structures and material innovation will provide continuous opportunities in this regard. Additionally, more mechanisms will be integrated into a single device to attain more comprehensive sensing functions. Finally, with regard to power supply, triboelectric sensors, piezoelectric sensors, thermo-electric sensors and pyroelectric sensors served as a feasible method. This is therefore the next cutting-edge frontier in personalized healthcare.
